# Screening of Cellular Stress Responses Induced by Ambient Aerosol Ultrafine Particle Fraction PM0.5 in A549 Cells

**DOI:** 10.3390/ijms20246310

**Published:** 2019-12-13

**Authors:** Pavlína Šimečková, Soňa Marvanová, Pavel Kulich, Lucie Králiková, Jiří Neča, Jiřina Procházková, Miroslav Machala

**Affiliations:** Veterinary Research Institute, Department of Chemistry and Toxicology, Hudcova 296/70, 62100 Brno, Czech Republic; simeckova@vri.cz (P.Š.); marvanova@vri.cz (S.M.); kulich@vri.cz (P.K.); kralikova@vri.cz (L.K.); neca@vri.cz (J.N.); prochazkova.j@vri.cz (J.P.)

**Keywords:** PM0.5, ultrafine particulate matter, p53, AhR, early stress response, unfolded protein response (UPR), inflammation, eicosanoids

## Abstract

Effects of airborne particles on the expression status of markers of cellular toxic stress and on the release of eicosanoids, linked with inflammation and oxidative damage, remain poorly characterized. Therefore, we proposed a set of various methodological approaches in order to address complexity of PM0.5-induced toxicity. For this purpose, we used a well-characterized model of A549 pulmonary epithelial cells exposed to a non-cytotoxic concentration of ambient aerosol particle fraction PM0.5 for 24 h. Electron microscopy confirmed accumulation of PM0.5 within A549 cells, yet, autophagy was not induced. Expression profiles of various cellular stress response genes that have been previously shown to be involved in early stress responses, namely unfolded protein response, DNA damage response, and in aryl hydrocarbon receptor (AhR) and p53 signaling, were analyzed. This analysis revealed induction of GREM1, EGR1, CYP1A1, CDK1A, PUMA, NOXA and GDF15 and suppression of SOX9 in response to PM0.5 exposure. Analysis of eicosanoids showed no oxidative damage and only a weak anti-inflammatory response. In conclusion, this study helps to identify novel gene markers, GREM1, EGR1, GDF15 and SOX9, that may represent a valuable tool for routine testing of PM0.5-induced in vitro toxicity in lung epithelial cells.

## 1. Introduction

Particulate matter (PM) in the ambient air is known to play an important role in the development of cardiovascular and respiratory diseases and is classified as carcinogenic to humans (Group 1; [1]). Especially ultrafine particles with an aerodynamic diameter (d_ae_) <100 nm could easily enter and transfer within organisms and interact with cells and subcellular components [2]. At the ultrastructural cellular level, aerosol particles were observed either enclosed in membrane vesicles and autophagosomes, or associated with mitochondrial membranes and lamellar bodies in A549 pulmonary cells [3]. In order to reveal the mechanism of PM-induced toxicity, in vitro studies in human airway cells have been performed using size segregated aerosol particles. Water suspension of fine particles PM2.5 were shown to induced oxidative stress, aryl hydrocarbon receptor (AhR)-mediated gene expression, inflammatory responses, DNA damage, changes in the cell cycle and autophagy in human bronchial and lung cells [4,5,6,7]. The ultrafine fraction was found to cause inflammatory, cytotoxic and genotoxic responses [8,9,10,11,12]. However, in addition to well-known effects, such as oxidative stress and inflammation, other mechanisms of nanoparticle toxicity were suggested and encompass endoplasmic reticulum stress (ER stress), dysfunction of lysosomes and dysfunction of autophagy [13,14,15]. Importantly, these intracellular events might be linked to cellular stress responses [16].

In this study, we used the PM0.5 fraction of size-segregated urban aerosol that was characterized previously [17] for the elemental composition and size distribution of single particles and their agglomeration under the experimental conditions. Surprisingly, both the ultrafine fraction (d_ae_ < 0.17 μm) and the lower accumulation fraction (LAF; 0.17 < d_ae_ < 0.5 μm) contained a similar type of particle with the d_ae_ < 0.5 μm, and which were prevalently nanosphere soot and carbonaceous particles with trace amount of N and S. The exception were metallic nanoparticles with Fe as adominating element, found only in the LAF. Additionally, the ultrasonic detachment of LAF from apolyurethane foam substrate had higher efficiency in contrast to the ultrafine fraction trapped on Teflon filter. Therefore, the LAF was chosen for the use in this in vitro study as the representative of PM0.5. We monitored PM0.5-induced cellular stress responses at the level of specific genes and protein expression. We examined the expression status of genes involved in AhR-mediated toxicity, genotoxicity and unfolded protein response (UPR) related to ER stress. Those included HSPA5 (heat shock protein family A member 5), DDIT3 (DNA damage inducible transcript 3) and XBP1s (spliced form of X-box binding protein 1). Besides, based on a recent data-mining study [18], we also evaluated expression of early stress response genes—early growth response 1 (EGR1), activating transcription factor 3 (ATF3) and growth differentiation factor 15 (GDF15)—that were suggested as relevant to stress response in lung cells [6,19,20,21,22] and thus can serve as new markers of toxicity. The expression of EGR1, ATF3 and GDF15 is induced by various stimuli, such as hypoxia, oxidative, genotoxic and ER stress, in various types of cells, and they are supposed to be involved in the maintenance of genetic integrity and cellular homeostasis. Furthermore, expression of these early stress response genes is activated prior to visible signs of cellular toxicity [18,23,24,25,26,27,28]. 

Additionally, changes in the levels of released arachidonic acid (AA) metabolites were examined by liquid chromatography–tandem mass spectrometry (LC–MS/MS) as they represent a functional readout of induction of oxidative stress and inflammation and/or anti-inflammatory response [29,30]. 

The aim of this study was to screen whether PM0.5, when tested at non-cytotoxic concentrations, induced various types of stress responses that were previously found to be modulated after the exposure to engineered nanoparticles and/or chemical stimuli, but that have not yet been studied in the case of airborne ultrafine fractions. We compared deregulation of UPR, autophagy and early stress response genes with activation of a set of genes involved among others in AhR and p53 signaling.

## 2. Results

In order to determine whether the aerosol particles smaller than 0.5 μm are able to induce cellular stress responses, pulmonary A549 cells were exposed to PM0.5 in a concentration of 25 μg/cm^2^ for 24 h, which was chosen according to the study Gualtieri et al. [3] and shown to be non-cytotoxic in the time frame of 72 h (WST-1 assay; data not shown). 

### 2.1. Intracellular Localization

**Figure 1:**
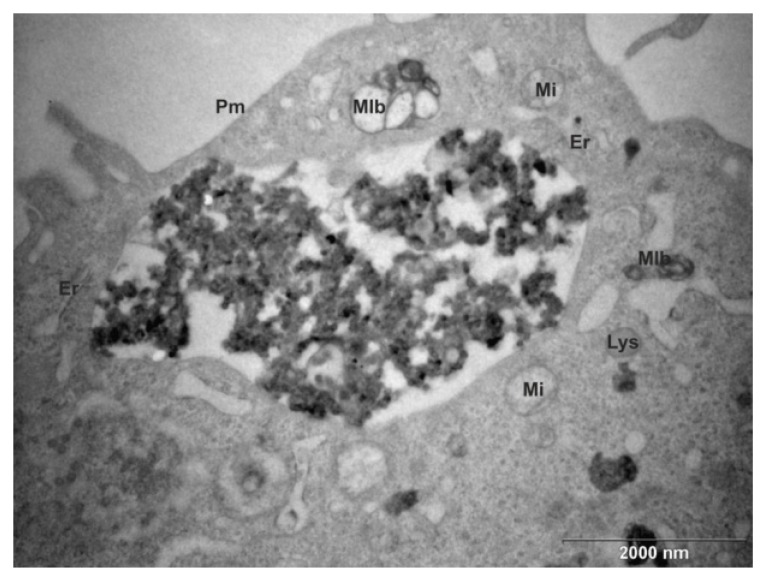
Transmission electron micrograph of ultrathin section of an A549 cell after 6 h exposure to PM0.5: Identification of PM aggregates inside a vesicle; Er, rough endoplasmic reticulum; Pm, cytoplasmic membrane; Mi, mitochondria; Mlb, multilamellar body; Lys, lysosome.

To ensure that aerosol particles were taken into the cells, ultrathin sections of A549 cells exposed to PM0.5 were examined by transmission electron microscopy. After 3 h of exposure, particles were not found on the ultrathin sections, but after 6, 12 and 24 h exposure, particles occurring in intracellular aggregates of various sizes were observed. They were engulfed by plasmatic membrane in some cases, while in other cases the membrane was not clear. Cellular membrane structures often occurred inside the vesicles with larger aggregates of particles, suggesting that the particles were deposited in multivesicular bodies or autophagosomes (Figure 1).

### 2.2. Expression Analysis of a New Set of Genes Involved in Various Signaling Pathways Including AhR and p53 

**Figure 2:**
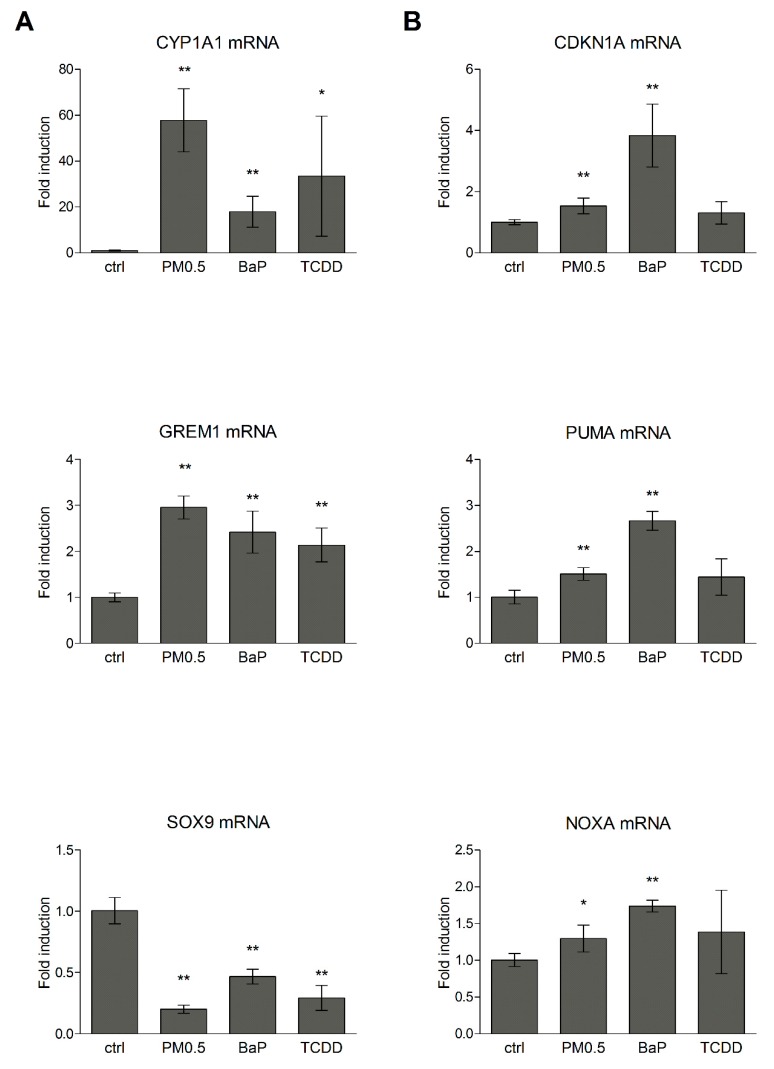
Induction of AhR–dependent genes (**A**) and DNA damage response genes (**B**). Relative mRNA levels of CYP1A1, GREM, SOX9, CDKN1A, NOXA, and PUMA were measured by qRT-PCR in A549 cells exposed to PM0.5 (in concentration 25 μg/cm^2^) for 24 h. A total of 10 nM TCDD and 1 μM benzo[a]pyrene (BaP) were used as positive controls for activation of AhR signaling and DNA damage response, respectively. The results are expressed as means ± SD of three independent experiments. The means are significantly different from the negative control (ctrl) at * *p* < 0.05 and ** *p* < 0.01.

Next, we examined the expression status of model genes associated with AhR (cytochrome P450 1A1; CYP1A1) and p53 (CDK1A, PUMA, NOXA) activation. Furthermore, gremlin 1 (GREM1) and SOX9 have been shown recently to have a similar 2,3,7,8-tetrachlorodibenzo-p-dioxin (TCDD)-like expression fingerprint during short-term adaptive response of A549 cells to both toxic exogenous and endogenous ligands, and may thus serve as markers of AhR-dependent transcriptional activity in lung epithelial cells [19]. Herein, we therefore included GREM1 and SOX9 expression analysis into testing of PM0.5-induced toxicity. PM0.5 exposure at non-cytotoxic concentration changed mRNA levels of AhR model target gene CYP1A1, as well as expression of proposed AhR lung-specific targets GREM1 (increased expression) and SOX9 (decreased expression, as shown in Figure 2A). 

**Figure 3:**
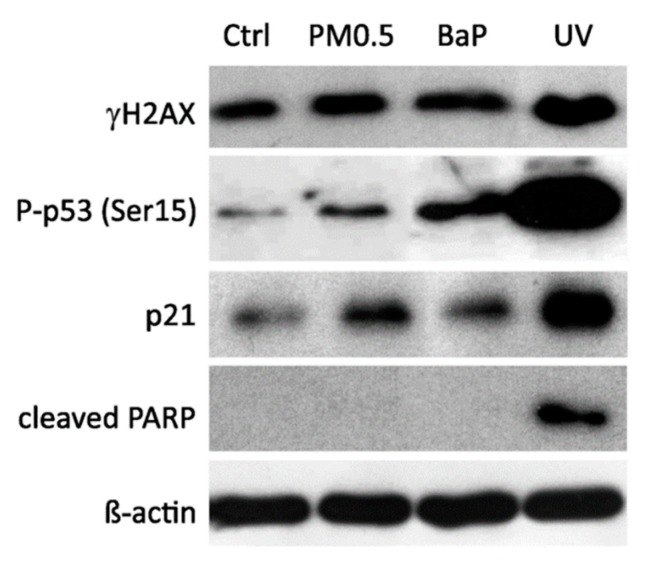
Western blot detection of proteins involved in DNA damage response and apoptosis. A549 cells were exposed to PM0.5 for 24 h and protein levels of phosphorylated H2AX (γH2AX), p53 phosphorylated on Ser15, p21, and cleaved PARP were detected. A total of 10 μM BaP and UV-C irradiation were used as positive controls. Ctrl, negative control. Detection of β-actin was used to confirm the equal loading.

DNA damage responses were identified in A549 cells on both mRNA and protein levels as previously described [31]. Weak genotoxic effects of PM0.5 were suggested by slightly but significantly induced expression of p53 transcriptional target genes, cyclin dependent kinase inhibitor 1A (CDKN1A, the gene encoding p21 protein), PUMA and NOXA, as determined by real-time quantitative qRT-PCR (Figure 2B). Moreover, the levels of phosphorylated histone H2AX protein (γH2AX), which is involved in cellular responses to DNA double-strand breaks and DNA replication stress [32], as well as levels of phosphorylated p53 (Ser15) and p21 proteins, were also elevated. Despite increased mRNA level of pro-apoptotic genes PUMA and NOXA, apoptosis was not massively induced by PM0.5 as confirmed by the absence of signal specific for cleaved PARP in western blots (Figure 3).

### 2.3. Induction of Novel Toxicity Markers—Early Stress Response Genes and Unfolded Protein Response

**Figure 4:**
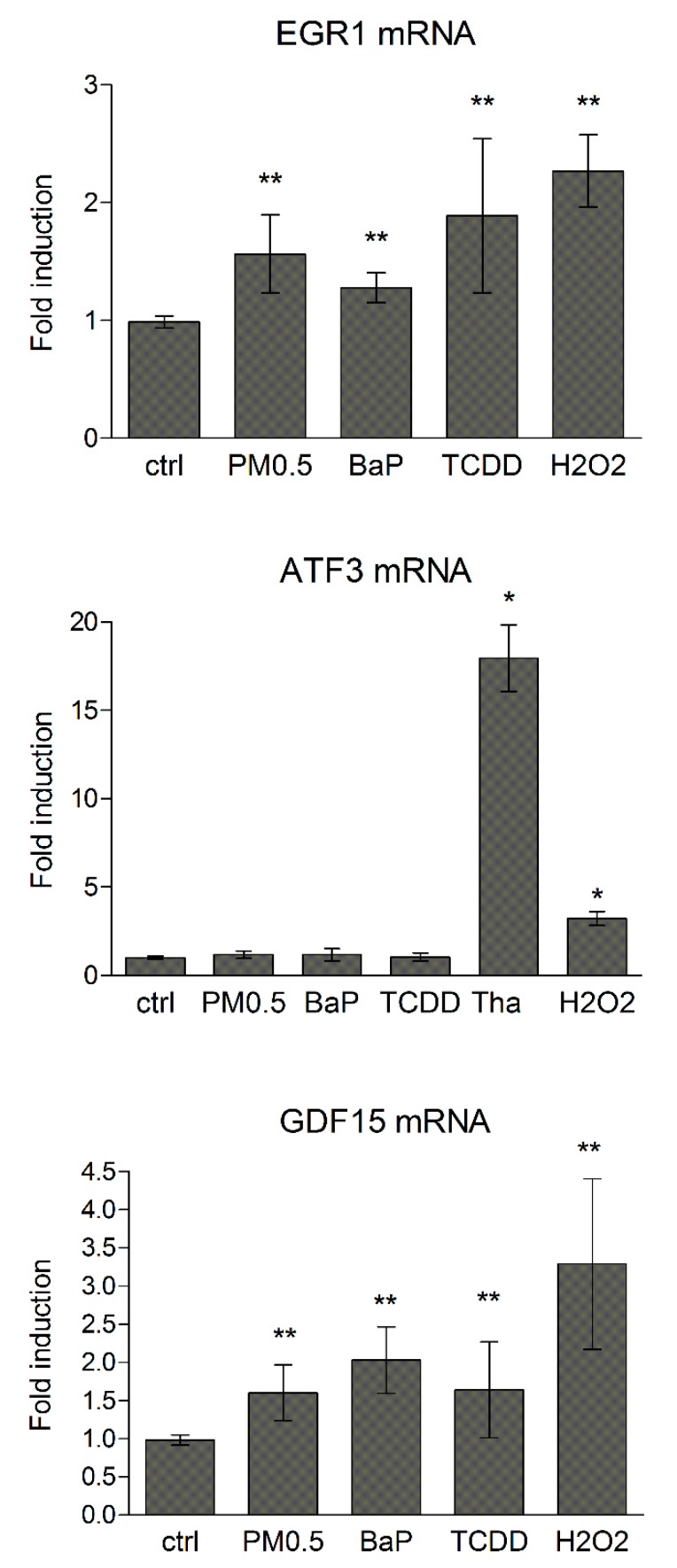
Induction of early stress response genes. EGR1, ATF3 and GDF15 mRNA following 24 h exposure of A549 cells to PM0.5, were determined by qRT-PCR. A total of 1 μM BaP, 10 nM TCDD, 100 nM thapsigargin (Tha) or 400 μM H_2_O_2_ were used as positive controls. The results are expressed as means ± SD of three independent experiments. The means are significantly different from the negative control (ctrl) at * *p* < 0.05 and ** *p* < 0.01.

Cellular response to various environmental stress conditions includes an activation of unspecific genes, mainly transcription factors that modulate expression of genes involved in maintenance of cellular homeostasis [22,23,24]. Our results show that 24h exposure of A549 cells to PM0.5 significantly induced the expression of early response genes EGR1 and GDF15 but not that of ATF3 (Figure 4).

**Figure 5:**
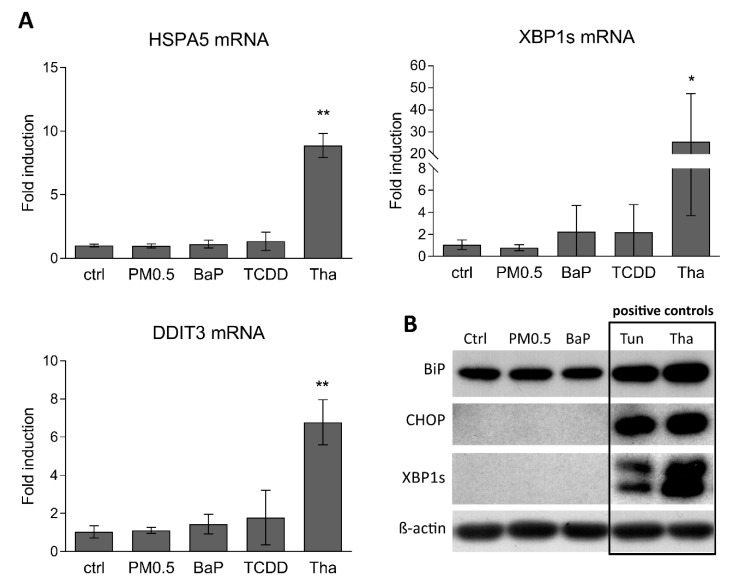
Induction of unfolded protein response. HSPA5 (BiP), DDIT3 (CHOP) and XBP1s mRNA (**A**) and protein (**B**) levels following 24 h exposure of A549 cells to PM0.5 in concentration 25 μg/cm^2^ were determined by qRT-PCR and western blotting, respectively. A total of 1 μM BaP, 10 nM TCDD, 100 nM thapsigargin (Tha) and 4 μg/mL tunicamycin (Tun) were used as positive controls. The results are expressed as means ± SD of three independent experiments. The means are significantly different from the negative control (ctrl) at * *p* <0.05 and ** *p* <0.01. Detection of β-actin was used to confirm equal loading.

ER stress leads to activation of genes involved in compensatory response, the UPR. We measured changes in HSPA5 (also known as binding immunoglobulin protein, BiP), DDIT3 (CHOP) and XBP1s mRNA and protein levels, representing activation of all three UPR pathways [33]. We found that, in contrast to commonly used ER stress inducers thapsigargin and tunicamycin, PM0.5 did not change mRNA levels of tested UPR-associated genes (Figure 5).

### 2.4. Autophagy

**Figure 6:**
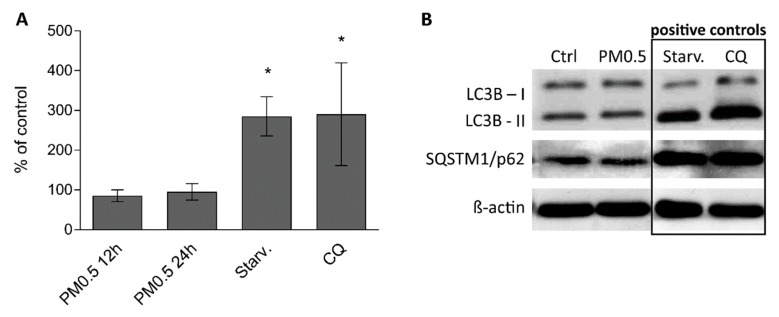
No autophagy was identified in A549 cells exposed to PM0.5. (**A**) Flow cytometric detection of autophagy using CYTO-ID^®^ staining in A549 cells. The percentage of autophagic cells was related to the negative control (100%). Starvation (Starv.) and 10 μM chloroquine (CQ) were used as positive controls. Neither 12 h nor 24 h exposure to PM0.5 increased autophagy in exposed cells. (**B**) Western blot detection of LC3B and SQSTM1/p62 protein levels in A549 cells exposed to PM0.5, CQ or starved for 24 h. Detection of β-actin was used to confirm equal loading. The means are significantly different from the negative control at * *p* <0.05.

To determine whether autophagy is increased after the exposure to aerosol particles, flow cytometry using a CYTO-ID^®^ Autophagy Detection Kit (Enzo Life Sciences, Farmingdale, NY, USA) and western blot detection of proteins LC3B (microtubule associated protein 1 light chain 3 beta) and sequestosome (SQSTM1/p62) were used. Starvation and exposure to chloroquine were used as positive controls. Flow cytometry did not reveal any increase in the percentage of autophagic cells after 12 or 24 h exposure as shown in Figure 6A. Similarly, levels of protein LC3B-II, a marker of autophagosomes, and SQSTM1/p62, which is a selective substrate for autophagy, were not elevated during 24 h exposure, as shown in Figure 6B.

### 2.5. Arachidonic and Linoleic Acid Metabolites

The concentrations of AA and linoleic acid metabolites were measured in cell culture medium harvested after 24 h exposure to PM0.5, namely prostaglandins (PGE_2_, PGA_2_, PGD_2_, PGF_2α_, PGF_2β_, 6-keto-PGF_1α_, i.e., a non-enzymatic product of PGI_2_, 8-iso-PGF_2α_, 15-keto-PGE_2_, 13,14-dihydro-15-keto-PGE_2_), hydroxyeicosatetranoic acids (5-HETE, 8-HETE, 11-HETE, 12-HETE, 15-HETE), and hydroxyoctadecadienoic acids (9-HODE, 13-HODE), thromboxane TxB_2_, and leukotriene LxA_4_. No significant differences in concentrations of the measured metabolites were found in a medium of control and exposed cells. The only exception was the significant change in the levels of PGD_2_ and 13,14-dihydro-15-keto-PGE_2_, the final metabolite of PGE_2_. Concentrations of several metabolites were under the detection limit (PGJ_2_, 9-HETE, 20-HETE, and the epoxyeicosatrienoic acids-8,9-EET, 11,12-EET, 14,15-EET). Concentrations of selected eicosanoids are shown in Table 1, the other data are not shown.

## 3. Discussion

Cellular stress responses are adaptive mechanisms that are activated to maintain homeostasis and survive adverse conditions [16]. Cellular stress responses are not as specific as, e.g., AhR-dependent CYP1A1 expression and are known to be induced by various stimuli; they represent integral parameters affected by different pathways linked to toxicity [34]. Modulation of cellular stress markers stands somewhere between determination of cytotoxicity/cell death and induction of specific biomarkers, such as CYP1A1 expression or DNA adduct formation. Therefore, they constitute an ideal first-line set of parameters suitable for identification of in vitro toxicity. Determination of stress markers in the cells exposed to xenobiotics such as drugs, environmental contaminants, or their mixtures is a novel approach in in vitro toxicity profiling [34,35,36]. 

In this study, we compared the expression profiles of selected cellular stress response genes (namely early response genes and UPR genes) with expression profiles of genes involved in multiple cellular processes, including AhR or p53 signaling in A549 cells exposed to non-cytotoxic concentrations of PM0.5. We observed expression changes in CYP1A1, SOX9 and GREM1 that were recently shown to be associated with activation of AhR signaling in lung epithelial cells [19] together with induction of p53-dependent transcriptional responses (monitored here by induced levels of both P-p53 and p21 protein and p53 target genes CDKN1A, NOXA and PUMA) [31]. The level of γH2AX protein, the marker of double strand breaks and/or DNA repair, was not significantly elevated, which together with the absence of PARP cleavage and unchanged mitochondrial activity even after 72 h exposure to PM0.5 (WST-1 assay, data not shown) suggests that PM0.5 at a concentration of 25 μg/cm^2^ does not induce apoptosis. Parts of genes, suggested here as positive markers of PM0.5 toxicity, includes early response genes, EGR1 and GDF15, that were previously shown to participate in adaptive response of cells to various stress stimuli including, e.g., ionizing radiation or viral infection [37,38]; both of them may be linked to AhR activation [39,40]. However, other transcription factor(s), for example p53, may be also involved in EGR1 and GDF15 induction after PM0.5 exposure [28]. EGR1 has been shown to be induced by cigarette smoke in lung cells, which again documents its association with AhR activation, and with chemokine production, MAPK activation, HSP70 signaling and with inflammatory responses [41,42,43,44]. GDF15 has been reported in the airway epithelium of smokers with chronic obstructive pulmonary disease and in human airway epithelial cells exposed to cigarette smoke [45]. A possible link between mixed AhR/p53 activation and induced levels of cellular stress markers EGR1 and GDF15 is also strongly supported by OTFBS (overrepresented transcription factor binding sites) analysis of human EGR1 and GDF15 genes (Table 2) as the binding site matrices for both transcription factors are significantly predicted in regulatory regions of GDF15 gene. In concordance with previously published data, showing EGR1 in human lung epithelial cells being a direct AhR target [39], OTFBS analysis did indeed confirm the prediction of DRE in regulatory regions of EGR1. Furthermore, the existence of AhR and p53 functional crosstalk has been suggested earlier [46,47,48]. It supports the hypothesis that various stress agents may activate simultaneously both signaling pathways and thus contribute to mixed induction of cellular stress marker expression. Besides, EGR1 may directly affect its own expression levels as well as levels of GDF15 as indicated by OTFBS analysis (Table 2). Finally, since EGR1 has been documented to bind regulatory regions of p53 promoter and to induce p53-dependent apoptosis, it is tempting to speculate that this early response gene is the key member of stress response circuit composed by AhR and p53. 

Surprisingly, another early response gene, ATF3, was not deregulated by PM0.5. ATF3 may be linked to genetic integrity and promotion of malignance of lung cells [24,49] and was identified as an AhR target gene in human liver HepaRG cells [40]. This is not consistent with recent finding that ATF3 is involved in PM-induced airway inflammation [21]. However, it was shown that ATF3 induction after DNA damage stimuli is only transient [50] and, moreover, it is associated with induction of apoptosis or with suppression of cell growth; these parameters were not observed as significantly changed in our study.

ER stress and autophagy are other possible mechanisms of nanoparticle-induced toxicity [7,14,15]. In our study, we did not detect any changes in mRNA or protein levels of UPR markers HSPA5 (BiP), DDIT3 (CHOP) and XBP1s [33]. Although we found PM0.5 localized in structures similar to autophagosomes or late endosomal multivesicular bodies, autophagy was not induced in the PM0.5-exposed A549 cells (as determined by LC3B and p62 protein level and CYTO-ID^®^ marker).

PGs, HETEs and HODEs, the oxidative products of AA and its precursor linoleic acid, are lipid mediators playing an important role in inflammation, atherosclerosis and carcinogenesis [51,52,53,54]. We detected no differences in the levels of AA, PGs, HETEs and HODEs released into the cell growth medium after the exposure of A549 cells to PM0.5. The only exception was the increase in PGD_2_ and the decrease in PGE_2_ metabolites, which may suggest a slight induction of anti-inflammatory response [30] at low, non-cytotoxic concentration of PM0.5. Importantly, we found no significant modulation of levels of 8-iso-PGF_2alpha_, 9- and 13-HODE, the markers of oxidative stress and lipid peroxidation [29,55], although oxidative stress is generally a well-accepted mechanism of nanoparticle-induced toxicity (e.g., [56]). 

In this study, various types of cellular stress responses—early stress response genes, UPR, autophagy and inflammation-related eicosanoids—were studied after the exposure of lung epithelial cells to fraction of PM0.5 in order to suggest novel readouts for testing PM0.5-induced toxicity. Moreover, the stress markers’ expression was compared with known AhR- and p53-dependent effects. We conclude that (1) the most sensitive events for assessment of exposure to low PM0.5 concentration are the activation of AhR- and p53- dependent pathways together with GREM1, EGR1 and GDF15 induction and that (2) chemical components adhered to the surface of nanoparticles (such as polycyclic aromatic hydrocarbons and other AhR agonists) are responsible for the observed adverse effects. This hypothesis has been already suggested previously [8,54] and is supported by studies on the effects of extractable organic matter from PM [57,58,59].

## 4. Materials and Methods 

### 4.1. Aerosol Collection

Ambient air aerosol particles were collected in the Prague city center in March 2015 by a high-volume cascade impactor (BGI-900; BGI Incorporated, Waltham, MA, USA) as was described in detail previously [17]. A lower accumulation fraction (0.17 < d_ae_ < 0.5 μm) of aerosol particles was collected on polyurethane foam impaction substrates. Besides the 49% of particles of relevant size, the fraction contained 37% of ultrafine particles (<0.17 μm) [17]. Therefore, lower accumulation fraction of aerosol particles was chosen as representative PM0.5 for in vitro experiments. 

### 4.2. Particle Separation

Aerosol particles of fraction D were separated from PUF substrates using a slightly modified procedure according to Gualtieri et al. [3]. Briefly, PUF substrates were placed into glass beakers with deionized MilliQ water and sonicated in an ultrasonic bath (Teson 10, 650 W; Tesla, Czech Republic) three times for 20 min. Each time the particle suspension was collected and pure MilliQ water was added to the substrate. The mass of the extracted particles was determined gravimetrically; particle concentration in water was 980 µg/mL. Particle suspension was aliquoted and stored in glass vials at −20 °C until use. Prior to in vitro experiments, a frozen aliquot of airborne particle suspension was thawed, vortexed and sonicated for 20 min in an ultrasonic bath, and then into the cell culture medium in concentration 25 μg/cm^2^ [17].

### 4.3. Cell Culture

Human lung carcinoma A549 cells were obtained from the American Type Culture Collection (ATCC, Rockville, MD, USA) and grown in Dulbecco’s Modified Eagle’s medium (D-MEM; Gibco, Thermo Fisher Scientific, Waltham, MA, USA) supplemented with 1.0 g/L glucose and pyruvate, 10% fetal bovine serum, 200 mM glutamine and 10 mg/mL gentamycin sulfate (Sigma-Aldrich, Prague, Czech Republic). The same medium supplemented with 10% fetal bovine serum was used also for the exposures. Cells were cultivated in plastic cell culture flasks (TPP, Trasadingen, Switzerland) in humidified atmosphere of 5% CO_2_ at 37 °C. 

### 4.4. Transmission Electron Microscopy

The confluent A549 cells, grown in plastic cell culture plates (22 cm^2^), were exposed to the PM0.5 suspension in concentration 25 μg/cm^2^ for 3, 6, 12 or 24 h. After the exposure, the cells were washed with Millonig buffer and harvested by scraping. The cells were fixed in 3% glutaraldehyde in Millonig buffer, post-fixed in 1% OsO_4_ solution in Millonig buffer, dehydrated in 30%, 50%, 70%, 90% and 100% aceton and embedded in Epon-Durcupan mixture (Epon 812, Serva, Heidelberg, Germany; Durcupan, ACM Fluka, Buchs, Switzerland). The ultra-thin sections (60–70 nm) were prepared using the Ultramicrotome Leica EM UC7 (Leica, Wien, Austria). The sections were stained with 2% uranyl acetate and 2% lead citrate and observed at 80 kV under a Philips EM 208 S Morgagni transmission electron microscope (FEI, Brno, Czech Republic). 

### 4.5. Real-Time Quantitative RT-PCR

The confluent A549 cells were exposed to the PM0.5 suspension in concentrations 25 μg/cm^2^ for 24 h. A total of 1 μM BaP (benzo[a]pyrene), 10 nM TCDD, 100 nM Tha or 400 μM H_2_O_2_ were used as positive controls. The cells were washed twice with PBS and harvested into the cell lysis buffer provided with the NucleoSpin RNA II Purification Kit (Macherey Nagel, Düren, Germany). Total RNA was isolated according to the manufacturer’s instructions. The levels of ATF3, CDKN1A, CYP1A1, DDIT3, EGR1, GDF15, GREM1, HSPA5, NOXA, PUMA, SOX9, and XBP1s mRNAs were determined by real-time RT-PCR using QuantiTect RT-PCR kit (Qiagen GmbH, Hilden, Germany). Sequences of primers and probes used for detection of GDF15, SOX9, GREM1, CYP1A1, and XBP1s were described previously [19,60,61]. The sequences of the other primers (all designed and synthesized by Generi Biotech, Czech Republic) and numbers of Universal Probe Library probes (UPL; Roche Life Sciences, Mannheim, Germany) are listed in Table 3. Hydroxymethylbilane synthase (HMBS; NM_000190) was used as the reference gene (predesigned qPCR assay, 3032-F, Generi-Biotech, Hradec Králové, Czech Republic). The RT-PCR reactions were carried out in 10 µL reaction mixture containing: 5 µl of QuantiTect Probe RT-PCR Master Mix, 0.1 µL of QuantiTect RT mix, 1.1 µL of solution of primers and probe, 1.8 µL of water and 2 µL of sample and were run on the LightCycler^®^ 480 System (version 1.5.0, Roche Diagnostics, Prague, Czech Republic) using the program published previously [19]. Changes in gene expression were calculated using the comparative threshold cycle method [62]. 

### 4.6. Overrepresented Transcription Factor Binding Site (OTFBS) Analysis

All OTFBS analyses were performed in Genomatix software (Matrix Library v9.1, Genomatix GmbH, Munich, Germany) using overrepresented transcription factor binding sites tool [63]. According to Genomatix recommendations, results were considered significant, when z-score values reached thresholds >2 or <−2.

### 4.7. Western Blotting

The A549 cells were seeded at an initial density 17,800 cells/cm^2^ and grown in 6-well cell culture plates for 72 h. After reaching confluency, the cell culture media was replaced by fresh one and the cells were exposed to the PM0.5 suspension in concentrations 25 μg/cm^2^ for 24 h. A total of 24 h exposure to BaP (10 μM), tunicamycin (4 μg/mL), thapsigargin (100 nM) and UV-C irradiation (UVG-11 lamp; UVP, Upland, CA, USA) were used as positive controls for the specific cellular process. The cells were harvested in lysis buffer (1% SDS, 100 mM Tris, 10% glycerol, protease inhibitor cocktail) and sonicated. Equal amounts of total protein were subjected to 10% SDS-polyacrylamide gel electrophoresis and transferred onto polyvinylidene fluoride membrane Hybond-P (GE Healthcare, Buckinghamshire, UK). The blotted membranes were blocked for 1 h in 5% non-fat milk and incubated with primary antibodies overnight at 4 °C. Following primary antibodies were used: LC3B (#3868), p21 (#2947), SQSTM1/p62 (#5114), BiP (#3177), XBP-1s (#83418), CHOP (#2895), phospho-histone H2A.X (Ser139; #2577), phospho-p53 (Ser15; #9284) antibodies (Cell Signaling Technology, Leiden, Netherlands). After washing, the membranes were incubated with secondary antibodies at room temperature for 1 h. Following secondary antibodies were used: peroxidase-conjugated anti-mouse A9044 (Sigma–Aldrich, USA) or #7076, anti-rabbit #7074 (Cell Signaling Technology, Leiden, Netherlands) or NA934 (GE Healthcare, USA). Expression of β-actin (A1978 antibody, Sigma-Aldrich, Danvers, MA, USA) was used to verify equal sample loading. To visualize peroxidase activity, ECL Plus reagent (GE Healthcare, Buckinghamshire, UK), or Luminata Forte Western HRP substrate (Millipore, Billerica, MA, USA) were used according to the manufacturer’s instructions. 

### 4.8. Flow Cytometry

The A549 cells were grown in 24-well cell culture plates. After reaching confluency, the cell culture medium was replaced by fresh one and the cells were exposed to the PM0.5 suspension in a concentration of 25 μg/cm^2^ for 12 or 24 h. Exposure to 10 μM chloroquine and starvation (cells without changing fresh medium) were used as positive controls. The increase in the percentage of autophagic cells was measured using a CYTO-ID^®^ Autophagy Detection Kit (Enzo Life Sciences, Farmingdale, NY, USA) according to the manufacturer’s instructions on a flow cytometer FACSCalibur (BD, Franklin Lakes, NJ, USA). The cells exposed to PM0.5 had high autofluorescence in FL1, FL2 and FL3 (data not shown). In order to avoid misinterpretation of the data, isocontrol fluorescence was subtracted from the fluorescence of samples with the CYTO-ID^®^ probe. 

### 4.9. Liquid Chromatography—Tandem Mass Spectrometry (LC-MS/MS)

After exposure of A549 cells to PM0.5, the cell culture medium was harvested and solid-phase extraction was used for extraction and purification of arachidonic acid metabolites using SELECT HLB SPE cartridges (Supelco, Sigma–Aldrich, St. Luis, MO, USA). Subsequently, the analytes were detected by LC–MS/MS using the Agilent 1200 chromatographic system (610 Triple Quad LS/MS, Agilent Technologies, Santa Clara, CA, USA) as published earlier [64]. All eicosanoid standards were purchased from the Cayman Chemical Company (Ann Arbor, MI, USA).

### 4.10. Statistical Analysis

The data were analyzed by Student’s *t*-test or Mann–Whitney tests. Differences were considered significant when *p* < 0.05. 

## 5. Conclusions

Effects of airborne particles on the expression status of markers specific for cellular toxic stress and on release of eicosanoids related to inflammation and oxidative damage are not yet characterized. Therefore, in this study, we analyzed the expression profiles of various cellular stress response genes that have been previously shown to be involved in, e.g., early stress response, endoplasmic reticulum stress, unfolded protein response and autophagy, and compared them with the expression profiles of genes dependent on aryl hydrocarbon receptor (AhR) or p53 signaling. We used a well-characterized model of pulmonary cells (A549) exposed to non-cytotoxic concentration of ambient aerosol particle fraction PM0.5 for 24 h. Effects of PM0.5 were compared with those induced by prototypical toxic compounds, including, e.g., BaP and TCDD. Our results show that only early stress response genes EGR1 and GDF15 were upregulated together with AhR- and p53-dependent genes and thus suggest the major role of both signaling pathways in PM0.5-mediated toxicity. The accumulation of PM0.5 was found in A549 cells, however, markers of ER stress, UPR or autophagy were not induced. Finally, extracellular release of prostaglandin D_2_ was also slightly increased. Based on our experimental data we can conclude that PM0.5 effects strongly resemble those elicited by BaP. Therefore, the induction of AhR-dependent and p53-dependent transcriptional activity (caused probably by polycyclic aromatic hydrocarbons and other AhR agonists bound to PM0.5) together with increased expression of cellular markers EGR1 and GDF15 are the most sensitive events, which may lead to adverse effects of PM0.5. 

## Figures and Tables

**Table 1:** Concentrations of selected eicosanoids. The cell growth medium was harvested after 24 h exposure of A549 cells to PM0.5. The concentrations of eicosanoids in the medium, measured by LC/MS–MS, are expressed as mean in pg/mL ± standard deviation. The experiment was done independently three times in technical duplicates. The data were analyzed by Student’s *t*-test. The mean of PGD_2_ and 13,14-dihydro-15-keto-PGE_2_ were significantly different from the negative control (Ctrl) at * *p* < 0.05. Abbreviations: PG, prostaglandin; Tx, thromboxane.

	PGE_2_	13,14-Dihydro-15-Keto-PGE_2_	PGD_2_	6-Keto-PGF_1α_	TxB_2_	8-Iso-PGF_2α_
Ctrl	9.1 ± 3.6	10.0 ± 3.5	15.6 ± 3.1	2.6 ± 1.1	74.4 ± 6.7	16.7 ± 4.2
PM0.5	9.4 ± 2.3	5.3 ± 1.8 *	23.7 ± 5.1 *	4.0 ± 1.1	65.2 ± 4.8	13.9 ± 5.6

**Table 2:** Overrepresented transcription factor binding sites (OTFBS) analysis. The results were considered significant, when z-score values reached thresholds >2 or <−2; TF, transcription factor.

TF Matrices Family	hEGR1	hGDF15
V$AHRR	2.12	2.04
V$P53F	–	4.24
V$EGRF	6.42	8.32

**Table 3:** Sequences of primers and numbers of UPL probes (Universal Probe Library; Roche Life Sciences, Germany) used in quantitative RT-PCR.

Gene Symbol/RefSeq Code		Sequences
CDKN1A (p21)	NM_00389.4	F: 5′-CCGAAGTCAGTTCCTTGTGG-3′ R: 5′-CATGGGTTCTGACGGACAT-3′ P: #82
DDIT3	NM_001195056.1 NM_001195054.1 NM_001195053.1 NM_001195055.1 NM_004083.5 NM_001195057.1	F: 5′-AAGGCACTGAGCGTATCATGT-3′ R: 5′-TGAAGATACACTTCCTTCTTGAACA-3′ P: #21
HSPA5	NM_005347.4	F: 5′-AGCCTGGCGACAAGAGTG-3′ R: 5′-TCCTTGGGCAGTATTGGATT-3′ P: #39
ATF3	NM_001206486.2 NM_001206488.2 NM_001040619.2 NM_001030287.3 NM_001674.3 NM_001206484.2	F: 5′-TTTGCCATCCAGAACAAGC-3′ R: 5′-CATCTTCTTCAGGGGCTACCT-3′ P: #53
EGR1	NM_001964.2	F: 5′-AGCCCTACGAGCACCTGAC-3′ R: 5′-GGTTTGGCTGGGGTAACTG-3′ P: #22
NOXA (PMAIP1)	NM_021127.2	F: 5′-GGAGATGCCTGGGAAGAAG -3′ R: 5′-CCTGAGTTGAGTAGCACACTCG -3′ P: #67
PUMA (BBC3)	NM_014417.3 NM_001127240.1	F: 5′-GACCTCAACGCACAGTACGA -3′ R: 5′-GAGATTGTACAGGACCCTCCA -3′ P: #68

## Abbreviations

AA	arachidonic acid
AhR	Aryl hydrocarbon receptor
ATF3	activating transcription factor 3
BaP	benzo[a]pyrene
BiP	binding immunoglobulin protein
CDKN1A	cyclin dependent kinase inhibitor 1A
CYP1A1	cytochrome P450 1A1
d_ae_	aerodynamic diameter
DDIT3	DNA damage inducible transcript 3
DNA	deoxyribonucleic acid
EGR1	early growth response 1
ER	endoplasmic reticulum
GDF15	growth differentiation factor 15
GREM1	gremlin 1
γH2AX	phosphorylated histone 2A family member X
HETE	hydroxyeicosatetranoic acid
HODE	hydroxyoctadecadienoic acid
HSPA5	heat shock protein family A member 5
IARC	International Agency for Research on Cancer
LAF	lower accumulation fraction
LC–MS/MS	liquid chromatography–tandem mass spectrometry
LC3B	microtubule associated protein 1 light chain 3 beta
OTFBS	overrepresented transcription factor binding sites
PARP	poly (ADP-ribose) polymerase-1
PG	prostaglandin
PM	particulate matter
qRT-PCR	quantitative reverse transcription polymerase chain reaction
SQSTM1	sequestosome 1
TCDD	2,3,7,8–tetrachlorodibenzo–*p*–dioxin
Tx	thromboxane
UPR	unfolded protein response
WHO	World Health Organization
XBP1s	spliced form of X–box binding protein 1
